# BPLT^+^: A Bayesian-based personalized recommendation model for health care

**DOI:** 10.1186/1471-2164-14-S4-S6

**Published:** 2013-10-01

**Authors:** Jiashu Zhao, Jimmy Xiangji Huang, Xiaohua Hu

**Affiliations:** 1 Information Retrieval and Knowledge Management Research Lab, York University, Toronto, ON, M3J1P3, Canada; 2 Department of Computer Science and Engineering, York University, Toronto, ON, M3J1P3, Canada; 3 School of Information Technology, York University, Toronto, ON, M3J1P3, Canada; 4 College of Information Science, Drexel University, Philadelphia, PA, 19104, USA

## Abstract

In this paper, we propose an Advanced Bayesian-based Personalized Laboratory Tests recommendation (BPLT^+^) model. Given a patient, we estimate whether a new laboratory test should belong to a "taken" or "not-taken" class. We use the bayesian method to build a weighting function for a laboratory test and the given patient. A higher weight represents that the laboratory test has a higher probability of being "taken" by the patient and lower probability of being "not-taken" by the patient. For the sake of effectiveness and robustness, we further integrate several modified smoothing techniques into the model. In order to evaluate BPLT^+ ^model objectively, we propose a framework where the data set is randomly split into a training set, a validation input set and a validation label set. A training matrix is generated from the training data set. Then instead of accessing the training data set repeatedly, we utilize this training matrix to predict the laboratory test on the validation input set. Finally, the recommended ranking list is compared with the validation label set using our proposed metric *CorrectRate_M_*. We conduct experiments on real medical data, and the experimental results show the effectiveness of the proposed BPLT^+ ^model.

## Background

Large amounts of clinic laboratory test data are collected and stored every day. Therefore, there is an increasing need for analyzing and utilizing the laboratory test data. The problem we are working on in this paper is to recommend laboratory tests for given patients. Health care recommendation problems have drawn researchers' attention for years. However, there are not a lot of studies conducted on the clinic laboratory test recommendation problem.

**Figure 1:**
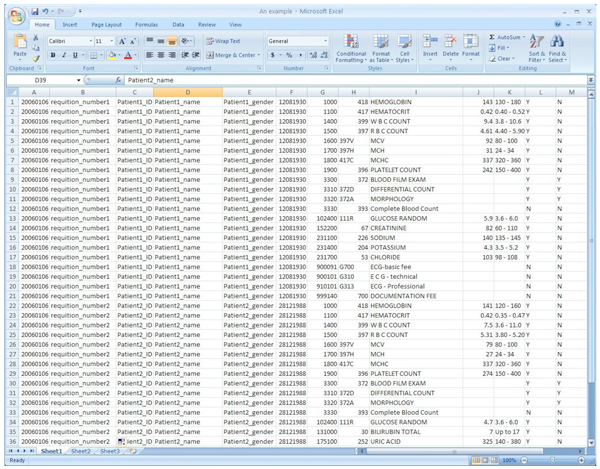
**An example dataset**. The format of the laboratory data sets is presented: the attributes from left to right are SDTE (SERVICE DATE), REQ# (REQUISITION NUMBER), PNUM (PATIENT HEALTH CARD#), PNAM (PATIENT NAME), PSEX (PATIENT SEX), BDTE (PATIENT DATE OF BIRTH), TSEQ (TEST SEQUENCE NUMBER), TEST (TEST CODE), DESC (TEST DESCRIPTION), RSLT (TEST RESULT), NORM (NORMAL RANGE), REXP (RESULT EXPECTED Y/N), EXRS (EXTENDED RESULT Y/N). The patient information in this table is fake due to privacy.

The medical data we are working on contains several years patients' laboratory test records. Figure 1 shows an example of the data format. Formally, the laboratory test prediction problem can be described as follows [1]: "Given a set of patients *P *= *{p*_1_, *p*_2_, ..., *p_n_} *and a set of laboratory tests *T *= *{test*_1_, *test*_2_, ... *test_M_}*, each patient *p_j _*has done tests *test*_*j*,1_, ..., *test_j,kj_*. If a doctor would like to assign a new test for patient *p_j_*, which test in T should be chosen?"

The computer systems have been playing for an important role in health care for years [2-8]. Statistic algorithms [9-12] lead an important role in investigating health care data. [13,14] extracts chemical keywords from a query patent by analyzing word frequency and the word's effect over the data collection. Bayesian learning is a widely used algorithm that shows good performance [15-19]. A semantic-based association rule mining approach is proposed to model the medical query contexts in [20]. Using a novel classifier based on the Bayesian discriminant function, Raymer, M. L. [21] present a hybrid algorithm that employs feature selection and extraction to isolate salient features from large medical and other biological data sets. Martín and Pérez [22] analyze the robustness of the optimal action in a Bayesian decision making problem in the context of health care. [23,24] studies the association between two words by simulating the impact of words in documents in the context of information retrieval. A probabilistic survival model is derived from the survival analysis theory for measuring aspect novelty of genomics data [25]. A mixture markov model is proposed to investigate user navigation patterns so that a personalized recommendation system for each user can be built [26]. In our previous work [1], we propose a laboratory test prediction model, which would objectively determine whether a laboratory test is associated to a patient. This paper is a significant extension to [1].

Smoothing [27] is a technique to create an approximating function that attempts to capture important patterns in the data, while leaving out noise or other fine-scale structures/rapid phenomena. The smoothing techniques have been used in many realms to improve the accuracy [28]. Based on the basic Bayesian algorithm and smoothing techniques, we propose an Advanced Bayesian-based Personalized Laboratory Tests recommendation (BPLT^+^) model, to investigate the correlation among laboratory tests for each patient. Evaluation is a crucial issue in the health care domain [29]. Some previous health care researchers do evaluation via patient interaction [30] or statistics [31]. We present a metric *CorrectRate_X _*by employing the idea of Mean Average Precision (MAP) [32] in Information Retrieval domain.

Four unique contributions are presented in this paper. Firstly, we learn the associations among laboratory tests and make personalized recommendations to patients without human interaction. Secondly, we integrate modified smoothing technologies to improve the personalized recommendation model and propose the BPLT+ model. Thirdly, we propose a framework to randomly generate a training data set, a validation input set and a validation label set. Fourth, we use a objective evaluation metric for personalized recommendation systems without patient interaction.

## Methods

### Bayesian-Based personalized laboratory tests recommendation (BPLT) model

Here we assume that the laboratory tests for a patient have associations among each other. For instance, if a patient is suspected to have diabetes, usually the doctor will assign both Hemoglobin test and Glucose Fasting test for this patient. We can see that there exists an association between Hemoglobin and Glucose Fasting with respect to some hidden information, diabetes in this case. On the other hand, if a patient is assigned Hemoglobin test, then it is very likely that this patient should also take Glucose Fasting test. In this section, we build a model for learning the associations of the laboratory tests, inferring the associations between patients and laboratory tests, and therefore recommending new laboratory tests to the patients. We regard the test recommendation problem as a special classification problem, where a test belongs to either a "taken" or "not-taken" class. We use Bayesian classifier as our basic classifier, and modify it to a personalized ranking model.

#### Basic concept: Bayesian classifier

A classification problem is the following [33]: given a set of training instances, each described with a set of n attributes and each belonging to exactly one of a certain number of possible classes, learn to classify new, unseen objects. In addition, each attribute has a fixed number of possible values. We use naive Bayesian classifier as our basic classifier in this paper, since it evaluates directly the probability of taking a test and the conditional probability among two tests. Moreover, naive Bayesian is easy to construct and has surprisingly good performance in classification, even though the conditional independence assumption is rarely true in real-world applications [34]. The probability model for a classifier is a conditional model

(1) $$\text{Pr}\left(C|F_{1},...,F_{n}\right)$$

where *F*_1_, ..., *F_n _*are attributes, and C is a class variable. By Bayesian criteria, it equals to

(2) $$\frac{\text{Pr}\left(C\right)\text{Pr}\left(F_{1},...,F_{n}|C\right)}{\text{Pr}\left(F_{1},...,F_{n}\right)}$$

The denominator is effectively constant, and the numerator is equivalent to the joint probability model

$$\begin{array}{l} \text{Pr}\left(C,\;F_{\text{1}},...,F_{n}\right)\\ =\text{ Pr}\left(C\right)Pr\left(F_{\text{1}}|C\right)\text{ Pr}\left(F_{\text{2}}|C,\;F_{\text{1}}\right)\text{ Pr}\left(F_{\text{3}}|C,\;F_{\text{1}},\;F_{\text{2}}\right)...\text{Pr}\left(F_{n}|C,F_{\text{1}},...,F_{n-\text{1}}\right) \end{array}$$

In naive Bayesian, it assumes the features are conditional independent

$$\text{Pr}\left(F_{i}|C,\;F_{j,0}\right)=\text{Pr}\left(F_{i}|C\right),\;for\;i\neq j$$

Therefore, the probability of a class C given feature *F*_1_, ..., *F_n _*is

(3) $$\text{Pr}\left(C|F_{1},...,F_{n}\right)=A\text{Pr}\left(C\right)\overset{n}{\underset{i=1}{\prod }}\left(F_{i}|C\right)$$

where $A=\frac{1}{\text{Pr}\left(F_{1},...,F_{n}\right)}$ is a constant.

#### The weighting function of BPLT model

In this Section, we describe the Bayesian-based Personalized Laboratory Tests recommendation (BPLT) model, which was proposed in our previous work [1]. More details are given in this paper. The purpose of BPLT model is to classify the laboratory tests for individual patients by their personal conditions. In the real world, it is often easier to obtain the patients' previous laboratory tests information. Therefore, the BPLT model recommends additional new laboratory tests to patients, given the previous laboratory tests that the patients have taken.

Suppose we have a set of M laboratory tests *T *= {*test*_1_, *test*_2_, ..., *test_M _*}, and a patient *p_j _*who has taken tests *T_j _*= {*test*_*j*_,_1_, ..., *test_j,kj _*} where *test_j,i _*∈ *T *for all 1 *≤ i ≤ k_j_*. We denote the events that tests in *T_j _*are taken by *p_j _*as *F_j_*,_1_, *F_j_*,_2_, ...*F_j,M _*. For example, if we have 7 tests in T, and *p_j _*has taken *test*_3_, *test*_5 _and *test*_7 _could be represented as (*F_j_*,_1_, *F_j_*,_2_, ..., *F_j_*,_7_) = (0, 0, 1, 0, 1, 0, 1). Bayesian Classifier is employed to evaluate the association between *p_j _*a new test *test*_0 _where *test*_0 _∈ *T *and *test*_0 _∉ *T_j_*. We use *F*_*j*,0 _to represent the event of *p_j _*should take *t*_0_, and $F_{j,0}^{c}$ to represent the event of *p_j _*should not take *t*_0_. By Formula (3), the probability of *F_j_*,_0 _given *F_j_*,_1_, *F_j,2_, ...F_j,M _*is

$$\text{Pr}\left(F_{j,0}|F_{j,1},F_{j,2},\ldots F_{j,M}\right)\propto \text{Pr}\left(F_{j,0}\right)\overset{M}{\underset{i=1}{\prod }}\text{Pr}\left(F_{j,i}|F_{j,0}\right)$$

The probability of $F_{j,0}^{c}$ given *F_j_*,_1_, *F_j_*,_2_, ... *F_j,M _*is

$$\text{Pr}\left(F_{j,0}^{c}|F_{j,1},F_{j,2},\ldots F_{j,M}\right)\propto \text{Pr}\left(F_{j,0}^{c}\right)\overset{M}{\underset{i=1}{\prod }}\text{Pr}\left(F_{j,i}|F_{j,0}^{c}\right)$$

In the BPLT model, we reward the tests with high probability of "taken" and low probability of "not-taken". The correlation between a new test *test*_0 _and a given patient *p_j _*is shown in Definition 1 [1].

**Definition 1 ***The correlation between a new test test*_0 _*and a given patient p_j _is defined as the log function of the probability of p_j _should take test *_0 _*divided by the probability of p_j _should not take test *_0 _*given F*_*j*,1 _, *F_j_*,_2_, ... *F_j,M_*.

(4) $$corr\left(test_{0},p_{j}\right)=\text{log}\frac{\text{Pr}\left(F_{j,0}|F_{j,1},F_{j,2},\ldots F_{j,M}\right)}{\text{Pr}\left(F_{j,0}^{c}|F_{j,1},F_{j,2},\ldots F_{j,M}\right)}$$

We can see that higher value of *corr*(*test*_0_, *p_j_*) indicates that *test*_0 _has more association with *p_j_*. The calculation of *corr*(*test*_0_, *p_j_*) can be further simplified as follows

(5) $$\begin{array}{l} corr\left(test_{0},p_{j}\right)\\ =\text{logPr}\left(F_{j,0}|F_{j,1},F_{j,2},\ldots F_{j,M}\right)-\text{log}\left(F_{j,0}^{c}|F_{j,1},F_{j,2},\ldots F_{j,M}\right)\\ =\text{logPr}\left(F_{j,0}\right)\overset{M}{\underset{i=1}{\prod }}\text{Pr}\left(F_{j,i}|F_{j,0}\right)-\text{logPr}\left(F_{j,0}^{c}\right)\overset{M}{\underset{i=1}{\prod }}\text{Pr}\left(F_{j,i}|F_{j,0}^{c}\right)\\ =\text{log}\frac{\text{Pr}\left(F_{j,0}\right)}{\text{Pr}\left(F_{j,0}^{c}\right)}+\overset{M}{\underset{i=1}{\sum }}\frac{\text{logPr}\left(F_{j,i}|F_{j,0}\right)}{\text{Pr}\left(F_{j,i}|F_{j,0}^{c}\right)} \end{array}$$

Moreover, a test either belongs to a "taken" class or a 'not taken" class. Thus, the following two formulas are held.

$$\begin{array}{c} \text{Pr}\left(F_{j,0}\right)+\text{Pr}\left(F_{j,0}^{c}\right)=1\\ \text{Pr}\left(F_{j,i}|F_{j,0}\right)\text{Pr}\left(F_{j,0}\right)+\text{Pr}\left(F_{j,i}|F_{j,0}^{c}\right)\text{Pr}\left(F_{j,0}^{c}\right)=\text{Pr}\left(F_{j,i}\right) \end{array}$$

from which we can obtain $\text{Pr}\left(F_{j,0}^{c}\right)$ and $\text{Pr}\left(F_{j,i}|F_{j,0}^{c}\right)$

$$\begin{array}{c} \text{Pr}\left(F_{{}_{j,0}}^{c}\right)=1-\text{Pr}\left(F_{j,0}\right)\\ \text{Pr}\left(F_{j,i}|F_{j,0}^{c}\right)=\frac{\text{Pr}\left(F_{j,i}\right)-\text{Pr}\left(F_{j,i}|F_{j,0}\right)\text{Pr}\left(F_{j,0}\right)}{1-\text{Pr}\left(F_{j,0}\right)} \end{array}$$

Thus $\text{Pr}\left(F_{j,0}^{c}\right)$ and $\text{Pr}\left(F_{j,i}|F_{j,0}^{c}\right)$ in (5) can be eliminated in *corr *(*test*_0_, *p_j _*), as shown below

$$\text{log}\frac{\text{Pr}\left(F_{j,0}\right)}{1-\text{Pr}\left(F_{j,0}\right)}+\overset{M}{\underset{i=1}{\sum }}\text{log}\frac{\text{Pr}\left(F_{j,i}|F_{j,0}\right)\left(1-\text{Pr}\left(F_{j,0}\right)\right)}{\text{Pr}\left(F_{j,i}\right)-\text{Pr}\left(F_{j,i}|F_{j,0}\right)-\text{Pr}\left(F_{j,0}\right)}$$

A joint probability for patient *p_j _*take both of the tests *test_i _*and *test*_0 _is

$$\text{Pr}\left(F_{j,i},F_{j,0}\right)=\text{Pr}\left(F_{j,i}|F_{j,0}\right)\text{Pr}\left(F_{j,0}\right)$$

The definition of the correlation between *test*_0 _and *p_j _*is

$$\begin{array}{l} corr\left(test_{0},p_{j}\right)\\ =\text{log}\frac{\text{Pr}\left(F_{j,0}\right)}{1-\text{Pr}\left(F_{j,0}\right)}+\overset{M}{\underset{i=1}{\sum }}\text{log}\frac{\text{Pr}\left(F_{j,i},F_{j,0}\right)\left(1-\text{Pr}\left(F_{j,0}\right)\right)}{\text{Pr}\left(F_{j,0}\right)\left(\text{Pr}\left(F_{j,i}\right)-\text{Pr}\left(F_{j,i},F_{j,0}\right)\right)}\\ =\left(k-1\right)\cdot \text{log}\frac{1-\text{Pr}\left(F_{j,0}\right)}{\text{Pr}\left(F_{j,0}\right)}+\overset{M}{\underset{i=1}{\sum }}\text{log}\frac{\text{Pr}\left(F_{j,i}|F_{j,0}\right)}{\text{Pr}\left(F_{j,i}\right)-\text{Pr}\left(F_{j,i}|F_{j,0}\right)} \end{array}$$

which leads to the following Definition 2 [1].

**Definition 2 ***The weighting function for a laboratory test test*_0 _*for a patient p_j _is the simplified correlation between test*_0 _*and p_j_*

(6) $$w\left(test_{0},p_{j}\right)=\left(k-1\right)\cdot \text{log}\frac{1-\alpha }{\alpha }+\overset{M}{\underset{i=1}{\sum }}\text{log}\frac{\beta_{j,i}}{\gamma_{j,i}-\beta_{j,i}}$$

where

$$\begin{array}{c} \alpha =\text{Pr}\left(F_{j,0}\right)=\frac{number\;of\;patients\;taken\;test_{0}}{number\;of\;patients}\\ \gamma_{j,\;i}=\text{Pr}\left(F_{j,i}\right)=\frac{number\;of\;patients\;that\;F_{j,i}\;holds}{number\;of\;patients}\\ \;\;\;\beta_{j,i}=\text{Pr}\left(F_{j,i}|F_{j,0}\right)=\frac{\text{Pr}\left(F_{j,i},F_{j,0}\right)}{\text{Pr}\left(F_{j,0}\right)}\\ =\frac{1}{\alpha }\frac{number\;of\;patients\;that\;both\;F_{j,0}\;and\;F_{j,i}\;holds}{number\;of\;patients} \end{array}$$

The new laboratory tests will be ranked in a list according to *w*(*test*_0_, *p_j _*) for a given patient *p_j_*. In the later section, we will present the evaluation environments for the laboratory test ranking list.

### An advanced model: BPLT^+^

To have a more robust and better performance model, we further propose an advanced model, BPLT^+^, by improving the BPLT model using several smoothing techniques. There are two reasons for smoothing BPLT. One reason is that smoothing is a way to deal with noise within the data. Another reason is to avoid the mathematically meaningless. When *test*^0 ^laboratory test has not been observed in the previous visits, which means *α *= 0, the first part of formula (6) will become an irrational number. Meanwhile, when the joint frequency of two laboratory tests is zero, which means *β_j_,_i _*= 0, the second part of (6) will become an irrational number. Therefore, we introduce smoothing technologies to further improve BPLT model.

#### Smoothing techniques

In statistics, smoothing [27] is a technique to create an approximating function that attempts to capture important patterns in the data, while leaving out noise or other fine-scale structures/rapid phenomena. The main purpose of smoothing in this paper is to assign a non-zero probability to the unseen tests and improve the accuracy of test probability estimation in general.

The smoothing techniques are discussed based on the following definitions of a conditional probability [28].

(7) $$\text{Pr}\left(t|p\right)=\frac{c\left(t;p\right)}{\sum_{t\in T}c\left(t;p\right)}$$

where c(t;p) is the count of a patient taking a test. Here are some commonly used smoothing methods. Since we have defined a ranking problem, which is similar to the problems in Information Retrieval (IR), we use some widely used smoothing methods in language model in IR. The general form of a smoothed model [35] is assumed to be the following:

(8) $$\text{Pr}\left(t|p\right)=\left\{\begin{array}{cc} \underset{t}{\text{Pr}}\text{(}t|p\text{)} & if\;test\;t\;is\;observed\\ \text{Pr}\left(t|C\right) & otherwise \end{array}\right)$$

where Pr_*t*_(*t|p*) is the smoothed probability of a test t given the patient with existing tests. Pr(*t|C*) is the probability of a test t given the whole data set.

A smoothing method may be as simple as adding an extra count to every test, which is called additive or Laplace smoothing, or more sophisticated as in Katz smoothing, where tests of different count are treated differently. Three representative methods that are popular and effective are:

• The Jelinek-Mercer method

(9) $$\underset{\lambda }{\text{Pr}}\left(t|p\right)=\left(1-\lambda \right)\text{Pr}\left(t|p\right)+\lambda \text{Pr}\left(t|C\right)$$

where *λ *is a balancing parameter ranges from 0 to 1.

• Bayesian Smoothing using Dirichlet Priors

(10) $$\underset{\mu_{0}}{\text{Pr}}\left(t|p\right)=\frac{c\left(t;p\right)+\mu_{0}\text{Pr}\left(t|C\right)}{\sum_{t\in T}c\left(t;p\right)+\mu_{0}}$$

where *µ*_0 _is a balancing parameter, and *µ*_0 _*>*0. The Laplace method is a special case of this technique.

• Absolute Discounting

(11) $$\underset{\delta }{\text{Pr}}\left(t|p\right)=\frac{max\;\left(c\left(t;p\right)-\delta ,0\right)}{\sum_{t\in T}c\left(t;p\right)}+\sigma p\left(t|C\right)$$

where *δ *∈ [0, 1] is a discount constant and *σ *= *δ|p|_u_/|p|*, so that all probabilities sum to one. Here *|p|_u _*is the number of unique terms in document d, and *|p| *is the total count of words in the documents.

#### BPLT^+ ^with smoothing techniques

There are two parts in formula (6) that need smoothing. The first one is the conditional probability *β_j_,_i _*= Pr(*F_j,i_*|F_*j*,0_). Its smoothed format is as follows:

• BPLT^+ ^with Jelinek-Mercer

(12) $$\beta_{j,i}^{\lambda }=\left(1-\lambda \right)\beta_{j,i}+\lambda \gamma_{j,i}$$

• BPLT^+ ^with dirichlet priors

(13) $$\beta_{j,i}^{\mu }=\frac{\beta_{j,i}+\mu \gamma_{j,i}}{1+\mu }$$

• BPLT^+ ^with absolute discounting

(14) $$\beta_{j,i}^{\delta }=\frac{max\left(c\left(t;p\right)-\delta ,0\right)}{\sum_{t\in T}c\left(t;p\right)}+\delta \gamma_{j,i}$$

In Jelinek-Mercer BPLT^+ ^and Absolute Discounting BPLT^+^, we use the existing smoothing method. The smoothing parameters *λ*, *δ *are within the range of [0, 1]. In Dirichlet Priors BPLT^+^, we modify the Dirichlet smoothing technique, by divide both the numerator and the denominator in (10) by $\sum_{t\in T}c\left(t;p\right)$, and normalize the parameter *µ *to the range of 0[1], where $\mu =\frac{\mu_{0}}{\sum_{t\in T}c\left(t;p\right)}$.

Another part in formula (6) needs smoothing is $\text{log}\frac{\alpha }{1-\alpha }$, which is a simple division that could be smoothed

via Laplace smoothing as

(15) $$\text{log}\left(\frac{\alpha +\theta }{1-\alpha +\theta }\right)$$

where *θ *is a tuning parameter ranges from 0 to 1.

## Evaluation environments

### Datasets

The datasets in our experiment are obtained from Alpha Global IT [1,36]. Alpha Corporate Group provides laboratory, medical clinic, commercial electronic medical record and practice management software. The data set contains 78 monthly patient's laboratory test results. Our experiments use 6 month results, containing 1,048,575 patients' records, as a key study. Thousands of patients' records and more than 400 laboratory tests are included in our experiments. The data format is the same as the example shown in Figure 1. Our data set contains real patients' information, such as health card ID, age, gender, date of visit, laboratory test ID, laboratory test results. We only use the patient ID and laboratory ID attributes in this paper, and analyze the associations among these laboratory tests. In our future work, we will incorporate more attributes in the laboratory recommendation model.

### Validation data and measure

To evaluate BPLT^+ ^models objectively, we divide the data set into three components: a training set, a validation input set, and a validation label set. The data set is firstly randomly split into a training set and a validation set. In this step, we split based on the patients and do not split the records from a same patient. Then for the validation set, we randomly remove one test t^* ^from each patient *p_j_*, and store the t^* ^in the validation label set. The ranked list returned by BPLT^+ ^will be compared with t^* ^for each patient. To measure such comparison and finally evaluate the effectiveness of BPLT^+^, we use the following defined *CorrectRate_X _*[1]. Suppose the returned laboratory ranking list is $L=t_{1,j}^{\prime },\ldots t_{l,j}^{\prime }$, *CorrectRate_X _*validates whether t^* ^appears in the top ranked tests. The measure is modified from Mean Average Precision (MAP) [32] evaluation metric.

**Definition 3 ***The CorrectRate_X _evaluates the accuracy of a laboratory tests prediction system. It is the number of patients with the desired (golden standard) test matching one of the top X tests generated by the system, divided by the total number of the patients*.

(16) $$CorrectRate_{X}=\frac{\sum_{j=1}^{n}TOP_{j,X}}{n}$$

where

$$TOP_{j,X}=\left\{\begin{array}{c} 1\;if\;t^{*}\;matches\;a\;test\;in\;\left\{{t^{\prime }}_{1,j},\ldots {t^{\prime }}_{X,j}\right\}\\ 0\;\;otherwise \end{array}\right)$$

n is the number of patients, X is a parameter indicating how many top tests are compared to the golden standard test t*, *which is set to be 1 or 3 in this paper*.

We present an example to show how the *CorrectRate_X _*evaluates the model in Table 1. Suppose the laboratory test sets includes 200 tests and there are 5 patients in the validation set. As we have introduced, the BPLT^+ ^model returns a ranked list for each patient. Here "*>*" represents that the weight of the left-side laboratory test is higher than the weight of the right-side laboratory test. In our example, 2 out of 5 patients have the desired test t^* ^ranked in the top 1 position of the list, then *CorrectRate*_1 _equals 0.4. And 4 out of 5 patients have t^* ^appears within the top 3 positions of the returned ranking list, then *CorrectRate*_3 _equals 0.8. We can see that the top 3 positions include the top 1 position, so the following statement is always true: *CorrectRate*_1 _*≤ CorrectRate*_3_.

**Table 1:** An example of *CorrectRate_X_*

	*t∗*	Recommendation list	*X *= 1	*X *= 3
*p*_1_	*test*_104_	*test*_104 _*> test*_5 _*> test*_40 _> ...	*TOP*_1,1 _= 1	*TOP*_1,3 _= 1
*p*_2_	*test*_30_	*test*_30 _*> test*_3 _*> test*_18 _> ...	*TOP*_2,1 _= 1	*TOP*_2,3 _= 1
*p*_3_	*test*_2_	*test*_95 _*> test*_2 _*> test*_34 _> ...	*TOP*_3,1 _= 0	*TOP*_3,3 _= 1
*p*_4_	*test*_95_	*test*_78 _*> test*_19 _*> test*_58 _> ...	*TOP*_4,1 _= 0	*TOP*_4,3 _= 0
*p*_5_	*test*_198_	*test*_92 _*> test*_134 _*> test*_198 _> ...	*TOP*_5,1 _= 0	*TOP*_5,3 _= 1
*All patients*	--	--	*CorrectRate*_1 _= 0.4	*CorrectRate*_3 _= 0.8

This example contains a validation set of 5 patients, their desired laboratory test t*, the recommendation list, and the corresponding evaluation results.

### BPLT^+ ^System Framework

**Figure 2:**
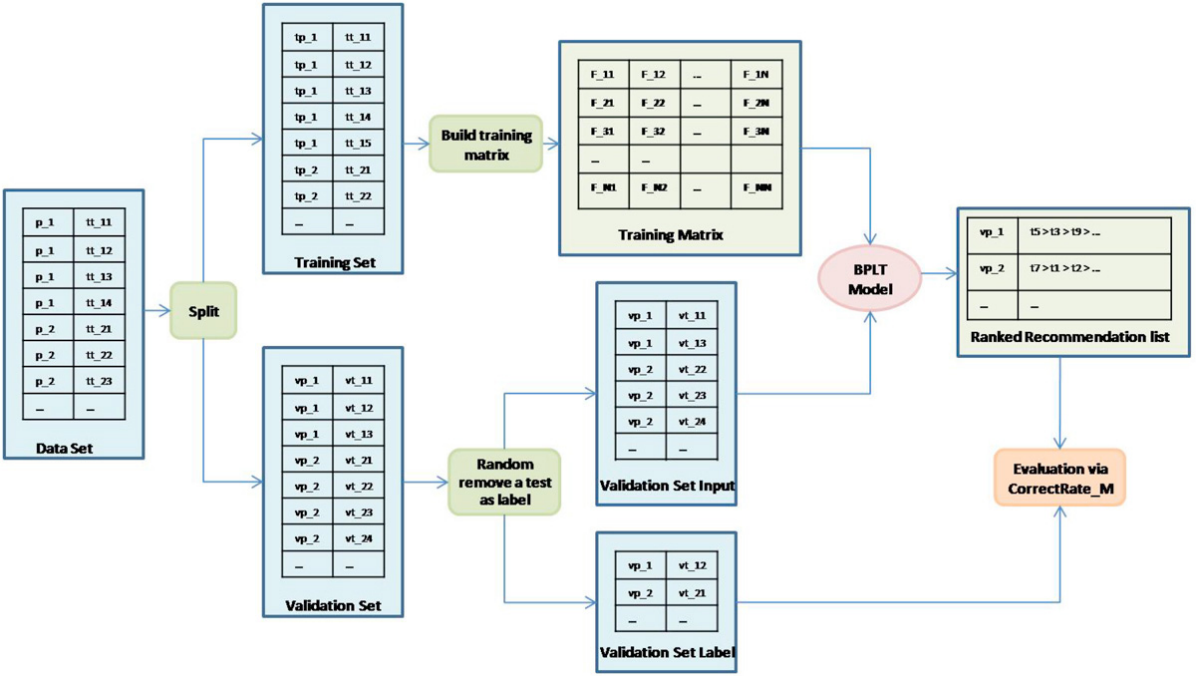
**BPLT^+ ^System Framework**. The procedures for processing the laboratory data and testing the BPLT^+ ^model are shown: (1) the rectangles represent the data sets; (2) the rounded rectangles present the implemented procedures; (3) the ovals show the personalized laboratory model; (4) the lines with arrows determine the directions through the framework.

The framework of BPLT^+ ^Model is shown in Figure 2. The data set in this framework is abstracted to contain only patient ID and laboratory test ID. The procedures in the proposed framework are described as follows.

• **Split**: First the data set is randomly split into a training set and a validation set.

• **Random Remove a test as label**: Since it is hard to objectively evaluate the performance of the BPLT^+ ^model, we further randomly remove a test for each visit of the patients from the validation set. These removed tests are regarded as labels of the validation set input. Our ultimate goal is to recommend the missing test for a patient's visit.

• **Build training matrix**: To avoid duplicate calculating the frequency of a test and the joint frequency between two tests, we build a training matrix out of the training data. This training matrix contains the frequency of co-occurrences of two laboratory tests. For example, if a patient in the training data did *test*_1 _and *test*_2 _together, then add 1 to *F*_12 _and *F*_21_. We can see that the training matrix is a symmetric matrix.

• **BPLT**^+ ^**model**: The correlation of a given *test*_0 _and a patient is calculated based on formula (6).

• **Evaluation via ***CorrectRate_X_*: Finally, the evaluation criteria *CorrectRate_X _*evaluates if the model made the correct recommendations.

## Results

We first show the overall performance under different training-validation proportion in Table 2[1]. We randomly take 40%, 50% and 60% of the data out of the raw data set as the training data and keep the rest as the validation data. In general, there is higher performance of BPLT^+ ^model on a larger training data set. This is because the larger training data set contains more information, and more knowledge can be learned. With the development of computer technology, larger amount of medical data will be available in practice. Therefore, we will use 60% of data as training data in the rest of this paper. As we have discussed before, *CorrectRate*_3 _is always higher than *CorrectRate*_1_. In general, the BPLT^+ ^model has promising performance with an accuracy of 0.7074 for *CorrectRate*_1 _and an accuracy of 0.7840 for *CorrectRate*_3_.

**Table 2:** Performance

Percentage of Training Data	*CorrectRate*_1_	*CorrectRate*_3_
60%	0.7074	0.7840
50%	0.6962	0.7837
40%	0.6823	0.7821

The overall performance of BPLT^+ ^with different training-validation proportions.

**Figure 3:**
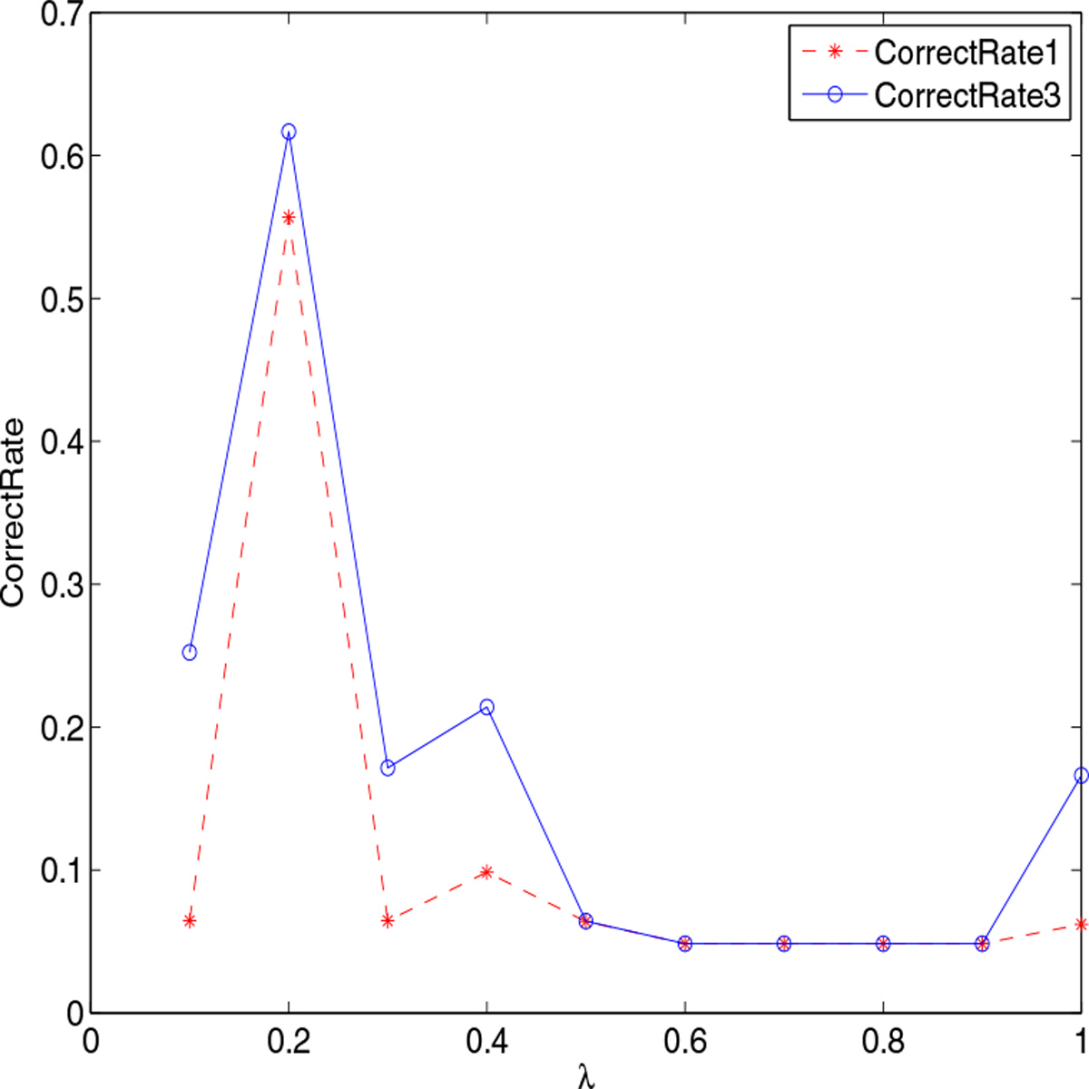
**Parameter Sensitivity of *λ *in Jelinek-Mercer BPLT^+^**. The influence of parameter *λ *is investigated: (1) the stars represent the performance of Jelinek-Mercer BPLT^+ ^under the evaluation metric *CorrectRate*_1_; (2) the circles represent the performance of Jelinek-Mercer BPLT^+ ^under the evaluation metric *CorrectRate*_3_; (3) *CorrectRate*_3 _is always higher than *CorrectRate*_1_; (4) Jelinek-Mercer BPLT^+ ^achieves its best performance when *λ *= 0.2.

**Figure 4:**
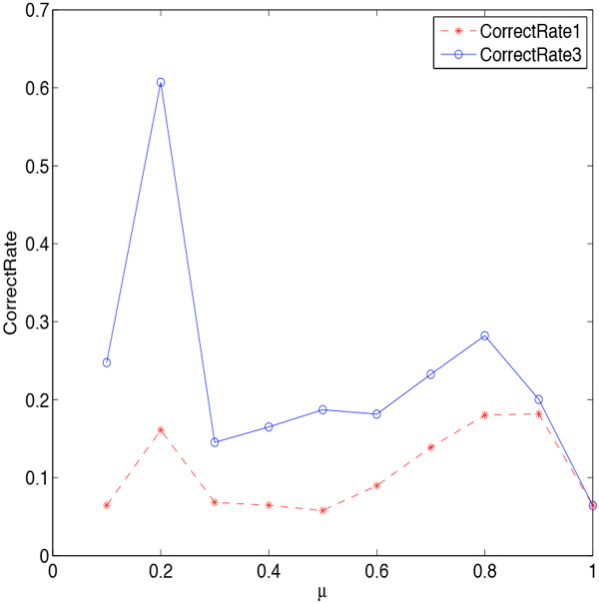
**Parameter Sensitivity of *µ *in Dirichlet Priors BPLT^+^**. The influence of parameter *µ *is studied: (1) the stars represent the performance of Dirichlet Priors BPLT^+ ^under the evaluation metric *CorrectRate*_1_; (2) the circles represent the performance of Dirichlet Priors BPLT^+ ^under the evaluation metric *CorrectRate*_3_; (3) Dirichlet Priors BPLT^+ ^achieves its best performance when *µ *= 0.2.

**Figure 5:**
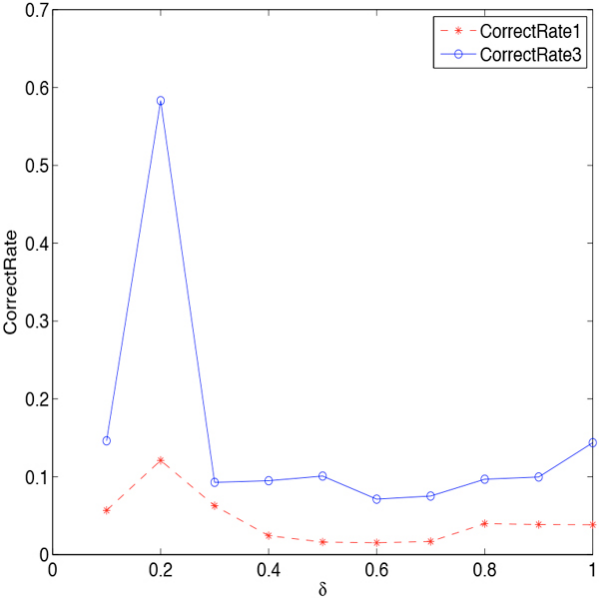
**Parameter Sensitivity of *δ *in Absolute Discounting BPLT^+^**. The influence of parameter *δ *is investigated: (1) the stars represent the performance of Absolute Discounting BPLT^+ ^under the evaluation metric *CorrectRate*_1_; (2) the circles represent the performance of Absolute Discounting BPLT^+ ^under the evaluation metric *CorrectRate*_3_; (3) Absolute Discounting BPLT^+ ^achieves its best performance when *δ *= 0.2.

Then we investigate how the smoothing parameters affect the effectiveness in detail. We first consider smoothing *β_j,i _*only. There are three smoothing technologies utilized to smooth *β_j,i_*. They are Jelinek-Mercer BPLT^+^, Dirichlet Priors BPLT^+ ^and Absolute Discounting BPLT^+^, with the corresponding parameters: *λ*, *µ*, *δ *∈ [0, 1]. We conduct experiments on these three methods individually. The change of *CorrectRate*_1 _and *CorrectRate*_3 _with respect to the parameters are shown in Figure 3, Figure 4, and Figure 5. We can see from the figures that the curve of *CorrectRate*_1 _is always below the curve of *CorrectRate*_3_, which is consistent as we have discussed Definition 3. With the increasing of parameters from 0.1 to 1, both *CorrectRate*_1 _and *CorrectRate*_3 _become higher at the beginning due to the incorporating of the smoothing portion. After reaching the maximum value, *CorrectRate*_1 _and *CorrectRate*_3 _become lower, since the weighing would tend to be more universal when too much smoothing is incorporated. All the smoothing parameters achieve their best performance at the value of 0.2. Comparing among these three methods, Jelinek-Mercer BPLT^+ ^obtains the best performance on both *CorrectRate*_1 _and *CorrectRate*_3_, which are 0.5569 and 0.6167. When it comes to the average value, Dirichlet Priors BPLT^+^'s average performance on *CorrectRate*_3 _is better than the other two, and Jelinek-Mercer BPLT^+^'s average performance on *CorrectRate*_1 _is the best.

**Figure 6:**
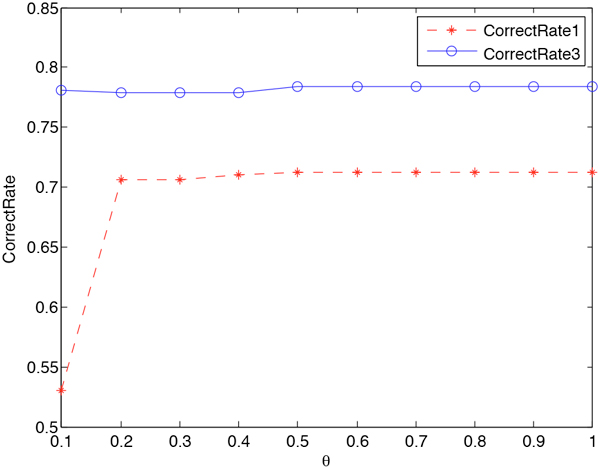
**Parameter Sensitivity of *θ***. The influence of parameter *θ *is presented: (1) we use the best smoothing technique for the first part in Formula 6, which is Jelinek-Mercer BPLT^+^; (2) the smoothing parameter *λ *is set to be optimal; (2) the stars represent the results of Jelinek-Mercer BPLT^+ ^under evaluation metric *CorrectRate*_1_; (3) the circles represent the results of Jelinek-Mercer BPLT^+ ^under evaluation metric *CorrectRate*_3_; (4) both metrics reach the maximum and tend to be stable when *θ *is greater than 0.5.

We further discuss to smooth the second part of (6), where the Laplace smoothing parameter is *θ*. As we have discussed before, Jelinek-Mercer BPLT^+ ^has the best performance on both *CorrectRate*_1 _and *CorrectRate*_3_. We focus on investigating the sensitivity of *θ *by fixing Jelinek-Mercer BPLT^+ ^with *λ *= 0.2. The results are shown in Figure 6. We can see that the *CorrectRate*_1 _increases while *θ *is increasing, and the *CorrectRate*_3 _decreases a little and then increases. Both of them reach the maximum and tend to be stable when *θ *is greater than 0.5.

## Conclusions and future work

An Advanced Bayesian based Personalized Laboratory Tests recommendation (BPLT^+^) model is proposed in this paper. Based on the assumption that hidden association could exist among laboratory tests, we employ a Bayesian approach to build a weighting function for scoring the correlation between a new laboratory test and a patient. To have a more robust and better performance model, we employ several enhanced smoothing technologies into the BPLT^+ ^model. The main purpose of smoothing in this paper is to assign a non-zero probability to the unseen laboratory tests and improve the accuracy of test probability estimation. We integrate existing smoothing techniques in the BPLT^+ ^model. In particular, we use three techniques, Jelinek-Mercer, Dirichlet Priors and Absolute Discounting approaches, to smooth the conditional probability of observing a patient taking an existing test when a new test *test*_0 _is given (Formula 12-14). Also we use Laplace method to smooth the log function in the BPLT^+ ^model (Formula 15). We conducted experiments to discuss the performance of the BPLT^+ ^model and the sensitivity of smoothing parameters. We find that BPLT^+ ^is able to make accurate recommendations under proper smoothing parameters.

Further, we propose a novel framework for effectively implementing BPLT^+ ^model and objectively testing personalized recommendation systems without human interactions, shown in Figure 2. Based on the real patients' laboratory test data, we randomly generate a training data set, a validation input set and a validation label set. A training matrix containing the laboratory test statistics is calculated from the training data set and stored. For new patients (the validation input set), instead of processing the original training set, we utilize this training matrix to predict the laboratory test on the validation input set, and compare the ranking results with the validation label set.

There are a few future directions of this research work. As we can see from the data format in Figure 1, we have not make use of all the attributes. In the future, we would like to conduct a comprehensive investigation for the patients' profiles. For example, we can cluster the patients into groups and investigate the similarities of the patients in the same group. We can also analyze the associations among laboratory test results and therefore further enhance our proposed personalized recommendation model. Moreover, we look forward to testing our proposed models in more real applications.

## Competing interests

The authors declare that they have no competing interests.

## Authors' contributions

JZ proposed BPLT^+ ^model, carried on the experiments and drafted the manuscript. JXH supervised the project and revised the manuscript. JXH also contributed in the study design and experiments. XH provides useful feedback. All authors read and approved the final manuscript.

## Declarations

The publication costs for this article were funded by the corresponding author.

This article has been published as part of *BMC Genomics *Volume 14 Supplement S4, 2013: Selected articles from the IEEE International Conference on Bioinformatics and Biomedicine 2012: Genomics. The full contents of the supplement are available online at http://www.biomedcentral.com/bmcgenomics/supplements/14/S4.

## References

[B1] Zhao J Huang JX Hu X Kurian J Melek W A Bayesian-based prediction model for personalized medical health care Bioinformatics and Biomedicine (BIBM), 2012 IEEE International Conference on: 4-7 October 2012 1 4 10.1109/BIBM.2012.6392623

[B2] Bates D Cohen M Leape L Overhage J Shabot M Sheridan T Reducing the frequency of errors in medicine using information technology Journal of the American Medical Informatics Association 2001 14 4 299 308 10.1136/jamia.2001.0080299 11418536 PMC130074

[B3] Ogiela L Tadeusiewicz R Ogiela M Cognitive techniques in medical information systems Computers in Biology and Medicine 2008 14 4 501 507 10.1016/j.compbiomed.2008.01.017 18339366

[B4] Shortliffe E Cimino J Biomedical informatics: computer applications in health care and biomedicine 2006 Springer

[B5] Melski J Geer D Bleich H Medical information storage and retrieval using preprocessed variables Computers and Biomedical Research, An International Journal 1978 14 6 613 10.1016/0010-4809(78)90038-1 738036

[B6] Thoma G Suthasinekul S Walker F Cookson J Rashidian M A prototype system for the electronic storage and retrieval of document images ACM Transactions on Information Systems 1985 14 3 279 291 10.1145/4229.4232

[B7] Frick S Uehlinger D Zenklusen R Medical futility: Predicting outcome of intensive care unit patients by nurses and doctors-A prospective comparative study* Critical Care Medicine 2003 14 2 456 461 10.1097/01.CCM.0000049945.69373.7C 12576951

[B8] Wu W Bui A Batalin M Au L Binney J Kaiser W MEDIC: Medical embedded device for individualized care Artificial Intelligence in Medicine 2008 14 2 137 152 10.1016/j.artmed.2007.11.006 18207716

[B9] Kajíc V Esmaeelpour M Považay B Marshall D Rosin P Drexler W Automated choroidal segmentation of 1060 nm OCT in healthy and pathologic eyes using a statistical model Biomedical Optics Express 2012 14 1 86 103 10.1364/BOE.3.000086 22254171 PMC3255345

[B10] Kokol P Pohorec S Štiglic G Podgorelec V Evolutionary design of decision trees for medical application Wiley Interdisciplinary Reviews: Data Mining and Knowledge Discovery 2012 14 3 237 254 10.1002/widm.1056

[B11] Pepe M The statistical evaluation of medical tests for classification and prediction 2004 Oxford University Press, USA

[B12] Rohian H An A Zhao J Huang X Discovering temporal associations among significant changes in gene expression Proceedings of IEEE International Conference on Bioinformatics and Biomedicine, IEEE 2009 419 423

[B13] Lupu M Huang XJ Zhu J TREC Chemical Information Retrieval - An Initial Evaluation Effort for Chemical IR Systems World Patent Information Journal 2011 14 3 248 256 10.1016/j.wpi.2011.03.002

[B14] Zhao J Huang X Ye Z Zhu J. York University at TREC 2009: Chemical Track Proceedings of the 18th Text REtrieval Conference 2009

[B15] Bernardo J Smith A Bayesian theory Measurement Science and Technology 2001 14 221 222

[B16] Chen J Huang H Tian F Tian S A selective bayes classifier for classifying incomplete data based on gain ratio Knowledge-Based Systems 2008 14 7 530 534 10.1016/j.knosys.2008.03.013

[B17] Clèries R Ribes J Buxo M Ameijide A Marcos-Gragera R Galceran J Martínez J Yasui Y Bayesian approach to predicting cancer incidence for an area without cancer registration by using cancer incidence data from nearby areas Statistics in Medicine 2012 10.1002/sim.4463 22237653

[B18] Huang X Hu Q A Bayesian Learning Approach to Promoting Diversity in Ranking for Biomedical Information Retrieval Proceedings of the 32nd Annual International Conference on Research and Development in Information Retrieval 2009 19 23

[B19] Liechty J Liechty M Muller P Bayesian correlation estimation Biometrika 2004 14 1 10.1093/biomet/91.1.1

[B20] Babashzadeh A Daoud M Huang J Using semantic-based association rule mining for improving clinical text retrieval Health Information Science 2013 186 197

[B21] Raymer M Doom T Kuhn L Punch W Knowledge discovery in medical and biological datasets using a hybrid bayes classifier/evolutionary algorithm IEEE Transactions on Systems, Man, and Cybernetics, Part B 2003 14 5 802 813 10.1109/TSMCB.2003.816922 18238233

[B22] Martín J Pérez C Muller P Bayesian robustness for decision making problems: Applications in medical contexts International Journal of Approximate Reasoning 2009 14 2 315 323 10.1016/j.ijar.2008.03.017

[B23] Hu Q Huang X Passage Extraction and Result Combination for Genomics Information Retrieval Journal of Intelligent Information Systems 2010 14 3 249 274 10.1007/s10844-009-0097-4

[B24] Zhao J Huang JX He B CRTER: using cross terms to enhance probabilistic information retrieval Proceedings of the 34th international ACM SIGIR conference, ACM 2011 155 164

[B25] Yin X Huang JX Li Z Zhou X A Survival Modeling Approach to Biomedical Search Result Diversification Using Wikipedia IEEE Transactions on Knowledge and Data Engineering 2013 14 6 1201 1212

[B26] Liu Y Huang JX An A Personalized recommendation with adaptive mixture of markov models Journal of the American Society for Information Science and Technology 2007 14 12 1851 1870 10.1002/asi.20631

[B27] Titterington D Common structure of smoothing techniques in statistics International Statistical Review/Revue Internationale de Statistique 1985 141 170

[B28] Zhai C Lafferty J A study of smoothing methods for language models applied to information retrieval ACM Transactions on Information Systems 2004 14 2 179 214 10.1145/984321.984322

[B29] Kononenko I Machine learning for medical diagnosis: history, state of the art and perspective Artificial Intelligence in Medicine 2001 14 89 109 10.1016/S0933-3657(01)00077-X 11470218

[B30] Donabedian A Evaluating the quality of medical care Milbank Quarterly 2005 14 4 691 10.1111/j.1468-0009.2005.00397.x 16279964 PMC2690293

[B31] Cook N Statistical evaluation of prognostic versus diagnostic models: beyond the ROC curve Clinical chemistry 2008 14 17 18024533 10.1373/clinchem.2007.096529

[B32] Sanderson M Information retrieval system evaluation: effort, sensitivity, and reliability Proceedings of the 28th annual international ACM SIGIR conference on Research and development in information retrieval, ACM 2005 162 169

[B33] Kononenko I Inductive and bayesian learning in medical diagnosis Applied Artificial Intelligence 1993 14 4 317 337 10.1080/08839519308949993

[B34] Zhang H Su J Naive bayesian classifiers for ranking Machine Learning: ECML 2004 501 512

[B35] Chen S Goodman J An empirical study of smoothing techniques for language modeling Computer Speech and Language 1999 14 4 359 394 10.1006/csla.1999.0128

[B36] Alpha Global IT http://www.alpha-it.com/

